# A study of the profiles of perceived inadequate qualifications and associated factors among nursing interns: a latent profile analysis

**DOI:** 10.3389/fpsyg.2026.1847171

**Published:** 2026-09-18

**Authors:** Fuqin Ouyang, Zhi hong Zhang, Lanfang Liu, Yan Zhang, Lu Zou, Can Bian

**Affiliations:** Nanchang Medical College, Nanchang, China

**Keywords:** clinical practice, nursing education, nursing interns, potential profile analysis, the sense of underqualification

## Abstract

**Purpose:**

To identify the latent profiles of nursing interns’ Perceived Inadequate Qualifications and to analyze their influencing factors.

**Methods:**

A convenience sample of 579 nursing interns from a medical college and a health vocational college in Nanchang, Jiangxi Province, China, was surveyed between May and September 2025. Data were collected Using a general information questionnaire, the Perceived Inadequate Qualifications Scale, psychological capital Questionnaire, Statistical analysis contained latent profile analysis, and unordered multinomial logistic regression analysis.

**Results:**

The Perceived Inadequate Qualifications of nursing interns were categorized into four types: “the Severe Type” (22.3%), “the Moderate Type” (45.4%), “the Mild Type” (22.5%), “Competent Qualification Type” (9.8%). Significant differences (*p* < 0.05) were observed across these four categories regarding reasons for choosing nursing as a profession, attitudes toward the nursing profession, occurrence of nursing errors during clinical practice, perceived psychological stress levels, satisfaction with knowledge and skills acquired during practice, ability to effectively integrate theoretical knowledge with clinical practice, and psychological capital.

**Conclusion:**

The Perceived Inadequate Qualifications of nursing interns exhibit heterogeneity.

**Implications for nursing education:**

Nursing educators and administrators should develop targeted clinical preceptorship models based on the latent profiles. They should promptly assess students’ professional competency and strengthen psychological support to mitigate feelings of underqualification.

## Introduction

Nursing care plays a vital role in patient care with the increasing demand for treatment and rehabilitation. With the aging of the global population and the increasing prevalence of chronic diseases (Cheng et al., 2020), nurses are expected to take more responsibilities in the future. By 2030, it is expected that there will be a shortage of 15 million medical professionals including nurses, especially in East Asia (Liu et al., 2017). China, one of East Asia’s countries, will have 4 nurses per 1,000 people, which is far lower than that of Western nations (Yang et al., 2025). Research indicates that nursing shortages are closely related to lower patient satisfaction and reduced quality of care (Chen et al., 2025). Nursing interns, as an essential human resource, play an important role in mitigating the gap in nursing human resources.

Perceived Inadequate Qualifications, as proposed by Sim and Lee (2017), refers to an individual’s subjective perception that their educational background, experience, knowledge, skills, or abilities fall short of the requirements of their job. The persistent stress of caregiving may also adversely affect caregivers’ physical and mental health and may consequently produce burnout and emotional exhaustion (Luitel et al., 2020). Nursing students do not have the knowledge and skills that nurses require, and they will encounter many difficulties and pressures in nursing work. Research has indicated that some clinical nurses currently experience incompetence due to insufficient academic qualifications and knowledge, which also serves as an independent factor influencing work avoidance behaviors among clinical nurses (Wang P. et al., 2024; Wang Y. et al., 2024). Among the nine studies that included nurses, estimates of the total annual attrition rate varied from 4.9% (the average from several African countries) to 44.3% in New Zealand (Castro Lopes et al., 2017). In China, studies also indicated that the attrition rate among nursing graduates, particularly those with bachelor’s degrees, was significantly higher at graduation than after entering the workforce (Jin et al., 2016).

Clinical practice is part of the curriculum in nursing education (Weng et al., 2025). In China, clinical internship refers to the supervised professional training conducted during the final year of undergraduate education, wherein students engage in clinical ability development under the guidance of clinical teachers in teaching hospitals (Zhou et al., 2024). The primary aim is to facilitate the translation of theoretical knowledge into professional competence. Lasting 12 months, this period is a critical component of clinical education and serves as an essential preparatory phase for nursing students transitioning to the role of registered nurses. Nursing students undergoing this training are referred to as nursing interns (Zeng et al., 2022). The researchers tended to be most concerned with the phenomenon of nursing internship because these interns encounter difficulties which, if not correctly managed, can adversely affect the students’ success and well-being. Certain research suggests that students are falling short of adequate clinical practice experience (Pasay-An et al., 2025). In China, the complex clinical environment, limited teaching approaches, specialized nature of the profession, and diverse interpersonal dynamics collectively impose significant physical and psychological stress on nursing students (Chen et al., 2025). Failure to address these challenges such as adapting to new ward environments, unfamiliar professional responsibilities, and anxiety may lead to reduced job motivation, diminished professional identity, and even decisions to leave the nursing profession after the internship (Hou et al., 2025). Research also shows that when individuals perceive their capabilities as insufficient to meet or manage their professional responsibilities, prolonged accumulation of such perceptions may lead to work avoidance behaviors (Zhang et al., 2024).

Stress coping theory (Lazarus and Folkman, 1984) proposes that when confronted with a stressor, an individual first engages in cognitive appraisal of the stressor or the external environment, evaluating factors such as harm, threat, and challenge. Based on this appraisal, and in consideration of their perceived capabilities, the individual adopts either positive or negative coping strategies, which in turn shape the eventual outcome. This process thus comprises four key components: the stressor, cognitive appraisal, coping, and outcome. Therefore, we can analyze why students with bachelor’s degrees may not engage in nursing work after graduation through the lens of this theory.

Previous studies have examined the relationship between insufficient nurse competence and withdrawal behaviors. One study assessed various factors and quantified four significant categories: teacher-related factors, patient-related factors, performance-related factors, and time/place-related factors that influenced student learning and practical training in a clinical setting. Some studies have explored latent profiles related to nurses’ work avoidance behaviors and work embedding, but their specific association with Perceived Inadequate Qualifications profiles remains unexamined in latent profile analyses. Therefore this study aims to explore the latent category characteristics of nursing interns’ perceived Inadequate Qualifications, examine the heterogeneity among categories, and investigate the influencing factors of different profiles. Latent profile analysis (Xu et al., 2023) adopts an individual-centered approach, grouping individuals with similar latent characteristics based on optimal fitting models when grouping variables are unclear. Compared to studies that categorize the Perceived Inadequate Qualifications solely based on overall scores, latent profile analysis fully accounts for internal heterogeneity within groups. This approach avoids potential issues where individuals may have identical total scores but exhibit distinct patterns of item-specific scores.

This study employed latent profile analysis to examine the current state of nursing interns’ Perceived Inadequate Qualifications and its influencing factors. The study aimed to provide a scientific reference for nursing education and nursing administrators in reforming and innovating student training processes, while also holding significant practical implications for nursing students’ career health development planning (Goh and Lopez, 2016).

## Aim of the study

To explore the latent class characteristics of nursing interns’ perceived Inadequate Qualifications based on latent profile analysis, to analyze the heterogeneity among different classes, and to explore the influencing factors of different profiles.

### Study design

A descriptive cross-sectional design was used to comply with the STROBE statement (Naleway et al., 2023).

### Participants

A total of 579 nursing graduates from a medical college and a health vocational college in Nanchang in 2025 were selected as the research participants by convenience sampling. Inclusion criteria: 1. The internship has been completed in strict accordance with the college’s internship syllabus; 2. Informed consent. Exclusion criteria: nursing graduates who did not complete the internship due to illness, dropout, etc. In this study, a total of 19 variables, according to the Kendall sample calculation method (Lin et al., 2025), the sample size should be 5–10 times the number of variables, and then consider 20% for the invalid questionnaires, the sample size range is 114–228 cases, while using Bayesian Information Criterion (Bayesian Information Criterion [BIC]) as the primary consideration of the selection model. The index sample size should be ≥ 200 (Kim, 2014). In this study, 579 nursing graduates were finally included.

### Data collection and quality control

We created the electronic questionnaire link containing the informed consent form through the independent “Questionnaire Star” platform to ensure anonymity and shared it in the WeChat group for nursing interns. Interested participants independently accessed the survey via the link shared in the WeChat group, reviewed the research information, provided informed consent, and subsequently completed the survey. A total of 624 questionnaires were distributed in this survey. Excluding the questionnaires with short response times and obvious illogical responses, 579 valid questionnaires were returned, with an effective recovery rate of 92.79%.

### Measures

The questionnaire and scale of this study include Socio-demographic characteristics, psychological capital Questionnaire and Perceived Inadequate Qualifications Scale. According to the reference, the author and the team members review and decide together.

### Socio-demographic characteristics

The general information survey reference materials are designed independently, including gender, age (years), education level, whether they are the only child, place of origin, reasons for choosing the nursing profession, whether there are medical staff in the family, teaching methods, educational background of the teaching instructor, attitudes of nursing staff towards the nursing profession, whether any nursing errors occurred during the internship, are you satisfied with the clinical internship environment? Do you feel significant psychological pressure? Are you satisfied with the knowledge and skills you learned during the internship? A total of 15 items on effectively combining theoretical knowledge with clinical practice during the clinical internship.

*Psychological Capital Questionnaire*: The Psychological Capital Questionnaire (PCQ) was developed by Luthans et al. (2007) and translated into Chinese by Luo and He (2010) and used to measure the level of psychological capital in nurses, and later applied to the group of nursing interns by other scholars. The questionnaire includes 4 dimensions with 20 items: hope (6 items), resilience (5 items), self-efficacy (6 items), and optimism (3 items). It uses a 6-point Likert scale, ranging from ‘strongly disagree’ to ‘strongly agree,’ scored from 1 to 6 points, with a total score range from 20 to 120 points. A higher score indicates a higher level of psychological capital (Luo and He, 2010). The Cronbach’s *α* coefficient for this scale was 0.923, and the Cronbach’s α coefficients for the various dimensions range from 0.718 to 0.890. In this study, the Cronbach’s α coefficient for this scale was 0.984.

### Perceived inadequate qualifications scale

The Perceived Inadequate Qualifications Scale was adapted by domestic expert Wang P. et al. (2024) and Wang Y. et al. (2024). This scale consists of 4 items and uses a 5-point Likert scoring method, where 1 point represents ‘completely disagree’ and 5 points represent ‘completely agree.’ The total score ranges from 4 to 20, with higher scores indicating a stronger sense of qualification insufficiency. The pre-trial Cronbach’s α coefficient for this scale was 0.777, and in this study, the scale’s Cronbach’s α coefficient was 0.941.

### Statistical design

#### Mplus 8.3

Latent profile analysis was conducted using Mplus 8.3 software (Meng et al., 2025) for LPA of the Sense of underqualification Scale; profiles were added step-by-step, and model fit was tested through indices. Evaluation indicators: ① The Akaike information criterion (AIC), Bayesian information criterion (BIC), and adjusted Bayesian information criterion (aBIC); the smaller the fit indices, the better the model fit; ② Information entropy is an evaluation metric for the accuracy of model classification, with values ranging from 0 to 1; the closer to 1, the higher the accuracy; ③ If the Lo–Mendell–Rubin likelihood ratio test (LMRT) and the bootstrap likelihood ratio test (BLRT) *p* values reach significance levels, it indicates that the K-class model fits better than the K − 1-class model (Elma et al., 2021). The above evaluation metrics only provide reference for profile decisions; when determining the best model, the interpretability of each profile category in practical work should also be considered.

#### SPSS 27.0

Statistical analysis was performed using SPSS 27.0 software (Zhou et al., 2026). Measurement data were expressed as mean ± standard deviation, and group comparisons were compared using analysis of variance; count data were presented as frequency, percentage, and rate, with group comparisons carried out using chi-squared tests or rank sum tests. The classification results of LPA were used as the dependent variable, while statistically significant variables from univariate analysis and psychological capital were treated as independent variables for multivariate logistic regression analysis, with a significance level of *α* = 0.05 (Thomas et al., 2022).

#### Ethical statement

The study was approved by the Ethics Committee of The First Affiliated Hospital of Nanchang Medical College (Ethics No. 2025(45)). All participating nursing interns gave informed consent before completing the electronic version of the questionnaire. The respondents’ personal information was kept strictly confidential, and their right to privacy was protected in accordance with the Declaration of Helsinki (Shephard, 1976).

## Results

### Socio-demographic characteristics

A total of 579 nursing interns were included in this study, including 529 females and 50 males, 165 younger than 20 years old, 244 junior college and 335 undergraduates, 524 non-only children. Place of birth: 374 from rural areas, 120 from urban areas, 85 were in towns; 264 voluntarily chose the nursing profession; 26.3% had medical staff at home, and 70.6% received diversified clinical teaching methods. The proportion of teachers with bachelor’s degree or above was 72.9%, the proportion of nurses with positive attitude towards nursing work was 65.5%, the proportion of nursing errors in the process of practice was 8.6%, the satisfaction of practice environment was 91.2%, the proportion of psychological pressure during practice was 80.3%, and the satisfaction of knowledge and skills learned in the process of practice was as follows: very dissatisfied 1.7%, dissatisfied 1.7%, generally satisfied 39%, satisfied 42%, very satisfied 15.6%, and the combination of theory and practice in clinical practice accounted for 93.4%, as shown in Table 1.

**Table 1:** Comparison of the distribution of potential profiles of the perceived inadequate qualifications among nursing interns with different socio-demographic characteristics.

Characteristic	Class 1 (*n* = 129)	Class 2 (*n* = 258)	Class 3 (*n* = 58)	Class 4 (*n* = 134)	chi-square	*p*
Sex						
Female	119(92.2)	240(93.0)	52(89.7)	118(88.1)	3.097	0.377
Male	10(7.8)	18(7.0)	6(10.3)	16(11.9)		
Reasons for choosing the nursing profession						
Voluntary	68(52.7)	103(39.9)	40(69)	53(39.6)	25.879	0.011
Parental Choice	44(34.1)	96(37.2)	12(20.7)	48(35.8)		
Teacher advice	0(0)	2(0.8)	1(1.7)	2(1.5)		
Assignment	11(8.5)	35(13.6)	3(5.2)	21(15.7)		
Admission	6(4.7)	22(8.5)	2(3.4)	10(7.5)		
Whether any nursing errors occurred during the internship						
Yes	1(0.8)	34(13.2)	3(5.2)	12(9.0)	17.749	<0.001
No	128(99.2)	224(86.8)	55(94.8)	122(91.4)		
Do you feel significant psychological pressure						
None	24(18.6)	38(14.7)	22(37.9)	30(22.4)	35.254	<0.001
Very light	22(17.1)	30(11.6)	13(22.4)	22(16.4)		
Fairly light	50(38.8)	99(38.4)	19(32.8)	43(32.1)		
Fairly heavy	31(24)	85(32.9)	4(6.9)	34(25.4)		
Extremely heavy	2(1.6)	6(2.3)	0(0)	5(3.7)		
Are you satisfied with the knowledge and skills you learned during the internship?						
Very dissatisfied	2(1.6)	4(1.6)	2(3.4)	2(1.5)	58.847	<0.001
Dissatisfied	3(2.3)	6(2.3)	0(0)	1(0.7)		
Generally satisfied	42(32.6)	125(48.4)	10(17.2)	49(36.6)		
Satisfied	69(53.5)	94(36.4)	21(36.2)	59(44)		
Very satisfied	13(10.1)	29(11.2)	25(43.1)	23(17.2)		
Effectively integrate theoretical knowledge with clinical practice during clinical internships.						
Yes	123(95.3)	230(89.1)	58(100)	130(97)	15.382	0.002
No	6(4.7)	28(10.9)	0(0)	4(3.0)		

Unit: persons (%). Only statistically significant variables are listed.

### Results of the perceived inadequate qualifications latent profile fitting for nursing interns

Using the scores for the four items related to inadequate qualifications among nursing interns as an external indicator, models with 1 to 5 categories were fitted. The model fitting results are shown in Table 2. As the number of profiles increased, the values of AIC, BIC, and aBIC exhibited a decreasing trend (Hosseinzadeh et al., 2021). When the 5-category model was included, the AIC, BIC, and aBIC values were minimal, but the probability share of one category was only 0.6%. With 4 categories, entropy reached its maximum. Combining AIC, BIC, and aBIC values, both LMR (*p* < 0.0047) and BLRT (*p* < 0.001) tests yielded statistically significant results (Wang et al., 2025). The model proved clear and concise, reasonably explaining the grouping patterns and meeting the criteria for LPA model adequacy. Therefore, this study ultimately selected the four-category model that fully explains the sense of inadequacy among nursing interns. The average probability of each category belonging to its latent profile was 96.3, 96.7, 98.5, and 99.3% (Tables 2, 3), indicating that the four-category model results are reliable and possess high discriminative power.

**Table 2:** Fit indices for latent profile analysis (*n* = 579).

Latent Profile	AIC	BIC	aBIC	LMR (p)	BLRT (p)	Entropy	Proportion of each profile
1-profile	6890.574	6925.465	6900.068	-	-	-	-
2-profile	5886.695	5943.392	5902.122	0.905	*P* < 0.001	*P* < 0.001	0.337/0.663
3-profile	5056.417	5134.92	5077.777	0.938	*P* < 0.001	*P* < 0.001	0.275/0.489/0.236
4-profile	4768.536	4868.846	4795.83	0.95	*P* < 0.0047	*P* < 0.001	0.223/0.446/0.100/0.231
5-profile	4670.721	4792.838	4703.949	0.957	*p* < 0.0129	*p* < 0.0145	0.104/0.237/0.440/0.007/0.212

AIC, Akaike Information Criterion; BIC, Bayesian Information Criterion; aBIC, adjusted BIC; LMR, Lo–Mendell–Rubin adjusted likelihood ratio test for K vs. K-1 profiles; BLRT, Bootstrapped Likelihood Ratio Test.

**Table 3:** Average assignment probability matrix for each potential category.

Classification	Class1	Class2	Class3	Class4
Class1	0.963	0.025	0.013	0.000
Class2	0.023	0.967	0.000	0.010
Class3	0.015	0.000	0.985	0.000
Class4	0.000	0.007	0.000	0.993

### Classification and naming of the perceived inadequate qualifications in nursing interns

The four items indicating inadequate qualifications among nursing interns show no overlap across categories and exhibit consistent morphological trends. Each potential profile category is named based on its scoring distribution across these items. Category 1: 129 participants (22.5%). These interns scored below average on all items related to feelings of inadequacy, indicating mild perceptions of inadequacy. Thus, this profile was named “Mild Feelings of Inadequacy.” Category 2 comprised 258 individuals (45.4%). Interns in this category scored at moderate levels across all items, termed “Moderate Competency Deficit.” Category 3 included 58 individuals (9.8%), with all items of competence anxiety significantly below the mean. This group was named “Competence-Competent,” indicating these interns could quickly master clinical work. Category 4 comprised 134 individuals (22.3%), with all items of competence anxiety significantly above the mean. This group was named “Competence Anxiety-Severe.”

### Distributional characteristics of latent profiles by sociodemographic characteristics among nursing interns

The results of the univariate analysis showed differences in the distribution of the latent profiles among nursing interns. The four groups of nursing interns demonstrated significant differences (*p* < 0.05) in the following areas: reasons for choosing nursing as a profession, attitudes toward the nursing profession, occurrence of nursing errors during internships, perceived psychological stress levels, satisfaction with knowledge and skills acquired during internships, and ability to effectively integrate theoretical knowledge with clinical practice during clinical placements, as shown in Table 1.

The results are shown in Table 4.

1. The score and latent profiles of the Perceived Inadequate Qualifications Scale.
2. The score and latent profiles of the Psychological Capital Questionnaire.

**Table 4:** The score and latent profiles of the perceived inadequate qualifications scale and the psychological capital questionnaire.

Scale	Core	C1 (*n* = 129)	C2 (*n* = 258)	C3 (*n* = 58)	C4 (*n* = 134)	H value	*P* value
Perceived inadequate qualifications	12 (9.14)	8 (8.35, 8.67)	12 (12.06, 12.27)	4 (4.25, 4.61)	16 (16.7, 17.32)	526.569	<0.001
List 1	3.04 ± 1.02	2 (2.3, 2.51)	3 (3.07, 3.19)	1 (1.11, 1.33)	4 (4.17, 4.35)	423.694	<0.001
List 2	2.80 ± 1.10	2 (1.9, 2.05)	3 (2.83, 2.96)	1 (0.98, 1.05)	4 (4.05, 4.29)	441.84	<0.001
List 3	3.01 ± 1.06	2 (2.13, 2.35)	3 (3.11, 3.22)	1 (1.07, 1.3)	4 (4.15, 4.36)	430.536	<0.001
List 4	2.85 ± 1.11	2 (1.83, 1.95)	3 (2.92, 3.03)	1 (1, 1)	4 (4.25, 4.41)	512.052	<0.001
Psychological capital	85 (75.100)	86.00 (82.69, 88.68)	79 (76.13, 79.9)	100 (90.23, 101.8)	100 (97.58, 103.54)	164.946	<0.001

### A multifactorial logistic analysis of factors influencing latent profiles of the perceived inadequate qualifications in nursing interns

Using category C4 as the reference group, categories C1, C2, and C3 as dependent variables, and statistically significant results from univariate analysis and psychological capital as independent variables, regression analysis was conducted. Independent variable assignment is shown in Table 5. Dummy variables were created for reasons for choosing the nursing profession. Statistically significant differences could be observed in psychological capital regarding the reasons for choosing nursing as a major, nursing staff attitudes toward the profession, occurrence of nursing errors during internships, perceived psychological stress levels, and satisfaction with knowledge and skills acquired during internships. Significant psychological pressure was a common risk factor for C1, C2, and C3 (*p* < 0.05). Moreover, psychological capital was a common risk factor for C1, C2, and C3 (*p* < 0.001) and exhibited a negative correlation. Reasons for choosing nursing as a major (*p* = 0.035, OR = 3.576) and occurrence of nursing errors during clinical practice (*p* = 0.004, OR = 23.714) were identified as specific risk factors for group C1. Attitude toward the nursing profession among nursing staff (*p* = 0.009, OR = 0.292) and satisfaction with knowledge and skills acquired during clinical practice (*p* = 0.04, OR = 0.279) were identified as specific risk factors for group C3, as shown in Table 6.

**Table 5:** Independent variable assignment.

Content	Variable assignment
Gender	male = 1; Female = 0
Age	<20 = 1; ≥ 20 = 2
Educational level	Specialty = 1; Undergraduate = 2
Only child	Yes = 1; No = 0
Place of birth	Rural areas = 1 Town = 2 Urban areas = 3
Reasons for choosing the nursing profession	Voluntary = 1; Parental Choice = 2; Teacher advice = 3; Assignment = 4; Admission = 5
Whether there are medical staff in the family	Yes = 1 No = 0
Teaching methods	Single = 1; Diversity = 2
Educational level of the teacher	Specialty = 1; Undergraduate = 2; graduate = 3
Attitudes of nursing staff towards the nursing profession	Very negative = 1; Somewhat negative = 2; Undecided = 3; Somewhat positive = 4; Very positive = 5
Whether any nursing errors occurred during the internship	Yes = 1, No = 0
Are you satisfied with the clinical internship environment	Very dissatisfied = 1; dissatisfied = 2; Generally satisfied = 3; satisfied = 4; Very satisfied = 5
Do you feel significant psychological pressure	None = 1; Very light = 2; Fairly light = 3; Fairly heavy = 4; Extremely heavy = 5
Are you satisfied with the knowledge and skills you learned during the internship	Very dissatisfied = 1; dissatisfied = 2; Generally satisfied = 3; satisfied = 4; Very satisfied = 5
Effectively integrate theoretical knowledge with clinical practice during clinical internships.	Yes = 1; No = 2
The Psychological Capital score	numeric value

Reasons for choosing the nursing profession has Setted the dummy variable.

**Table 6:** Multifactorial analysis of factors influencing potential profiles of the perceived inadequate qualifications in nursing interns (logistic regression).

Profiles	Variables		B	SE	Wald chi-square	P	OR	95%CI
C1 vs. C4 (C4 as a Reference)	Constant		1.371	1.784	0.59	0.442		
	Psychological capital		−0.075	0.01	53.575	*P* < 0.001	0.928	0.909, 0.947
	Reasons for choosing the nursing profession	Voluntary	1.274	0.605	4.439	0.035	3.576	1.093, 11.698
		Admission(reference)						
	Whether any nursing errors occurred during the internship	No	3.166	1.107	8.183	0.004	23.714	2.709, 207.541
		Yes(reference)						
	Do you feel significant psychological pressure	Fairly heavy = 4	2.048	1.029	3.958	0.047	7.75	1.031, 58.269
		Extremely heavy = 5 (reference)						
C2 vs. C4 (C4 as a Reference)	Constant		6.829	1.319	26.794	*P* < 0.001		
	Psychological capital		−0.093	0.01	91.587	*P* < 0.001	0.911	0.894, 0.928
	Do you feel significant psychological pressure	None = 1	1.912	0.874	4.781	0.029	6.764	1.219, 37.531
		Very light = 2	1.947	0.882	4.879	0.027	7.011	1.245, 39.469
		Fairly light = 3	2.206	0.841	6.871	0.009	9.076	1.745, 47.22
		Fairly heavy = 4	2.364	0.853	7.678	0.006	10.636	1.998.5, 6.628
		Extremely heavy = 5 (reference)						
C3 vs. C4 (C4 as a Reference)	Constant		−14.534	1.669	75.86	*P* < 0.001		
	Psychological Capital		−0.054	0.012	19.54	*P* < 0.001	0.948	0.926, 0.971
	Attitudes of nursing staff towards the nursing profession	Somewhat positive = 4	−1.231	0.473	6.767	0.009	0.29	0.115, 0.738
		Very positive = 5 (reference)						
	Do you feel significant psychological pressure	None = 1	19.017	0.686	768.512	*P* < 0.001	181487760.2	47308848.834, 696229308.413
		Very light = 2	19.242	0.684	792.279	*P* < 0.001	227461554.8	59566146.666, 868593350.707
		Fairly light = 3	19.14	0.626	933.82	*P* < 0.001	205308853.3	60154096.929, 700729084.328
		Fairly heavy = 4	18.144	0	-	-	75861327.18	75861327.184, 75861327.184
		Extremely heavy = 5 (reference)						
	Are you satisfied with the knowledge and skills you learned during the internship	Generally satisfied = 3	−1.275	0.621	4.214	0.04	0.279	0.083, 0.944
		Very satisfied = 5 (reference)						

C4 as reference.

## Discussion

Findings from this study indicated that nursing students’ perceived inadequacy scores during clinical placements were at a moderately high level. The perceived inadequacy score among nursing interns was 12 (9.14), higher than the results reported by Wang P. et al. (2024) and Wang Y. et al. (2024). This discrepancy may largely stem from differences in the study populations. As interns primarily assume trainee roles in clinical settings, their work remains in a learning phase. During clinical placements, students confront new environments, social dynamics, demanding workloads, and intensive academic requirements, collectively imposing significant psychological stress on nursing interns (Abaribe et al., 2025). Compounded by limited work experience, they are prone to medical errors and incidents, leading to diminished confidence in their nursing abilities. Most students perceive difficulties in performing their duties competently. These findings underscore the need for enhanced collaboration between academic institutions and clinical settings to thoroughly analyze learning challenges during internships and assist students in overcoming these difficulties. Simultaneously, educators should innovate teaching methods and approaches specifically targeting these learning obstacles (Saleh et al., 2025).

### The perception of inadequate qualifications among nursing students can be categorized into four types, with significant group heterogeneity observed

#### The perceived inadequate qualifications-mild accounted for 22.5%

Nursing students voluntarily chose the nursing major and demonstrated a relatively positive attitude toward the profession. Are they satisfied with the knowledge and skills acquired during clinical practice? (Highly Satisfied) This group primarily featured a high proportion of students who effectively integrated theoretical knowledge with clinical practice during internships (Taks et al., 2024). These nursing students independently chose their preferred specialty and actively combined theoretical knowledge with clinical practice during internships, solidifying their professional foundation. Their knowledge and skills genuinely improved, enabling them to quickly adapt to clinical job requirements in future work and meet societal demands.

#### The “moderate incompetence perception” (45.4%)

Typically characterized by choosing the major after complying with their own parents, average satisfaction with clinical learning knowledge and skills, and feeling significant psychological pressure during internships. This large group has considerable room for improvement in both knowledge mastery and skill proficiency. It is recommended that instructors promptly focus on this student cohort, employing diverse teaching methods and approaches to encourage proactive, in-depth learning and skill enhancement.

#### The “competent” category accounted for 9.8%

This group primarily consisted of high-achieving students who excelled academically and maintained a positive learning attitude. During clinical internships, they adapted quickly to the clinical environment, studied diligently, integrated theory with practice, and demonstrated the highest level of recognition for the nursing profession among clinical staff. They expressed high satisfaction with the knowledge and skills acquired in clinical settings and could effectively manage internship-related stress. These students are immediately job-ready upon graduation. Nursing administrators should pay special attention to this group. These students represented the elite among new nurses, possessing strong professional capabilities and the potential to serve as role models for their peers. The National Nursing Development Plan (2021–2025) states that by 2025, China’s total nursing workforce should reach 5.5 million (The National Health Commission of the People's Republic of China, 2022).

### Students experiencing “severe feelings of inadequacy” accounted for 22.3%

Compared to other categories, this group exhibited relatively higher rates of nursing errors. This may stem from their perception of being unprepared for clinical responsibilities. Clinical preceptors should intensify skill training, provide more hands-on practice opportunities, and enhance psychological support (Terefe et al., 2023). This requires a hands-off yet watchful approach, ideally implementing a one-on-one mentoring model.

### Analysis of the impact of psychological capital on nursing interns’ perceived inadequacy

PsyCap has a positive relationship with mental health in youth (Preston et al., 2023). Results indicated that psychological capital served as a risk factor for novice nursing students’ feelings of inadequacy, meaning higher levels of psychological capital correlated with a greater likelihood of being classified as competent (OR = 0.948). Nurses with higher psychological capital also exhibited higher levels of mental health (Zheng et al., 2025). Analysis indicated that such nursing students not only study diligently but also conscientiously complete clinical tasks assigned by instructors. They actively communicate with patients at the bedside and wholeheartedly assist instructors in fulfilling work responsibilities. Concurrently, nursing students with high psychological capital exhibited a more positive work attitude, performed nursing duties better, and enhanced their professional confidence. This suggests that nursing educators should proactively monitor students’ psychological states during clinical instruction, provide timely professional guidance and emotional support for emerging challenges, and regularly conduct mental health education training using clinical case studies. Such approaches help students understand and enhance their psychological capital, strengthen professional competence, and mitigate feelings of inadequacy (Dong et al., 2025).

## Conclusion and study implications

In summary, the perceived inadequate qualifications of nursing students during clinical practice can be categorized into four levels: “mild,” “moderate,” “competent,” and “severe.” Statistically significant differences were observed in psychological capital across these categories regarding the reasons for choosing nursing as a profession, nursing staff attitudes toward the field, occurrence of nursing errors during practice, perceived psychological stress levels, and satisfaction with knowledge and skills acquired during clinical training. Tables 1–6 and Figure 1 clearly illustrate the specific issues present among different categories of students, providing a scientific basis for nursing education and reform.

**Figure 1:**
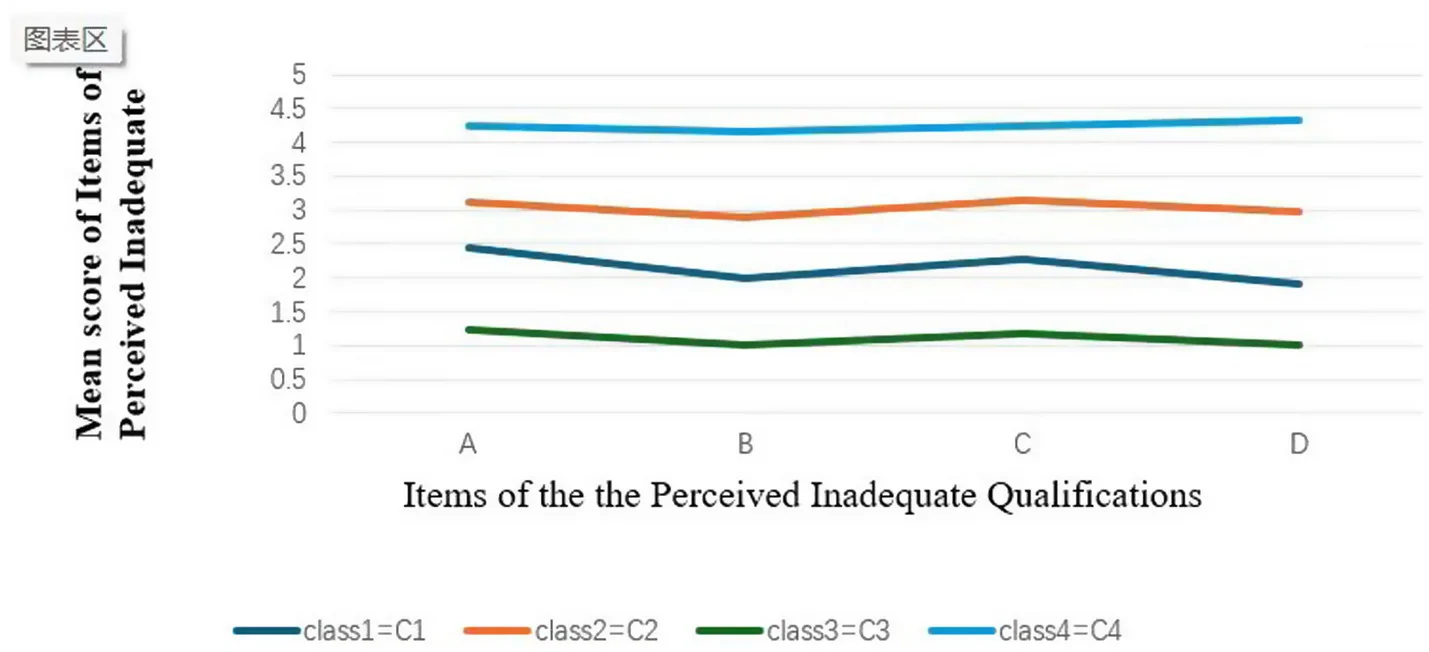
Response to each item in the 4 potential profiles of nursing interns perceived.

### Study limitations

The data in this survey were collected from only two medical schools, resulting in a relatively narrow sample scope that lacked generalizability. The sample range should be expanded to avoid information bias affecting the classification accuracy of potential profile analyses. More comprehensive and objective data should be used to reflect nursing students’ circumstances, thereby enhancing the reliability of research findings.

## Data Availability

The original contributions presented in the study are included in the article/supplementary material, further inquiries can be directed to the corresponding author.
